# Mass Balance and Drug Interaction Potential of Intravenous Eravacycline Administered to Healthy Subjects

**DOI:** 10.1128/AAC.01810-18

**Published:** 2019-02-26

**Authors:** Joseph V. Newman, Jian Zhou, Sergey Izmailyan, Larry Tsai

**Affiliations:** a Tetraphase Pharmaceuticals, Inc., Watertown, Massachusetts, USA

**Keywords:** ADME, drug interaction, eravacycline

## Abstract

Eravacycline is a novel, fully synthetic fluorocycline that is approved for the treatment of complicated intra-abdominal infections (cIAI) in adult patients. We report results from three studies in healthy subjects that investigated the distribution, metabolism, and excretion of intravenous (i.v.) eravacycline and the effect of a CYP3A4 inhibitor (itraconazole) and inducer (rifampin) on the pharmacokinetics (PK) of i.v. eravacycline. In the mass balance study, the majority of total radioactivity from [^14^C]eravacycline was recovered in the feces, suggesting biliary/fecal elimination is the major route of excretion for eravacycline and its metabolites after IV administration. The volume of distribution (217 liters) was greater than that of extracellular fluid, which suggests distribution beyond the central compartment. In the drug-drug interaction studies, mean area under the concentration-time curve from 0 h to the last time point (AUC_0_*_–t_*) and half-life were increased approximately 30% to 40% after a concomitant dose of i.v. eravacycline and itraconazole and clearance (CL) was decreased. A reduction in total eravacycline exposure (AUC) of approximately 25% to 35% and an increase in CL of approximately 50% occurred with concomitant eravacycline and rifampin treatment. The dose of eravacycline should be increased to 1.5 mg/kg of body weight every 12 h when coadministered with a strong CYP3A inducer.

## Abstract (precis)

Eravacycline is a novel, fully synthetic fluorocycline that is approved for the treatment of complicated intra-abdominal infections (cIAI) in adult patients. We report results from three studies in healthy subjects that investigated the distribution, metabolism, and excretion of intravenous (i.v.) eravacycline and the effect of a CYP3A4 inhibitor (itraconazole) and inducer (rifampin) on the pharmacokinetics (PK) of i.v.

## INTRODUCTION

Eravacycline is a novel, fully synthetic fluorocycline antibiotic that retains activity against the two major acquired tetracycline-specific resistance mechanisms: ribosomal protection and active drug efflux (1, 2). Eravacycline was developed for the treatment of serious bacterial infections caused by a broad spectrum of Gram-negative and Gram-positive aerobic and anaerobic pathogens, including multidrug-resistant organisms; *Enterobacteriaceae* that produce extended-spectrum β-lactamases, AmpC β-lactamases, and/or carbapenemases; and carbapenem-resistant Acinetobacter baumannii. In addition, eravacycline is highly active against both hospital- and community-acquired methicillin-susceptible or methicillin-resistant Staphylococcus aureus strains, vancomycin-susceptible or vancomycin-resistant Enterococcus faecium and Enterococcus faecalis, and penicillin-susceptible or penicillin-resistant isolates of Streptococcus pneumoniae.

Eravacycline was recently approved in the U.S. and the E.U. for the treatment of complicated intra-abdominal infections (cIAI) in adults, and the recommended dose regimen is 1 mg/kg of body weight every 12 h (q12h) for 4 to 14 days by intravenous (i.v.) infusion over 60 min.

In healthy volunteers given i.v. eravacycline at 1 mg/kg q12h for 10 days, the mean peak plasma concentration (*C*_max_) and area under the concentration-time curve from 0 to 12 h (AUC_0–12_) were 1,825 ng/ml and 6,309 ng·h/ml, while volume of distribution at steady state (*V*_ss_) and clearance (CL) were 4.0 liters/kg and 0.16 liters/h/kg (3). These values are consistent with other results from pharmacokinetic studies of i.v. eravacycline treatment (4, 5). Protein binding of eravacycline to human plasma proteins increases with increasing plasma concentrations, with 79% to 90% bound at plasma concentrations ranging from 100 to 10,000 ng/ml (6). In double-blind, randomized clinical studies, i.v. eravacycline treatment demonstrated efficacy and tolerability in patients with cIAI (7, 8).

Eravacycline is metabolized into three compounds: the C-4 epimer TP-498 and the metabolites TP-6208 and TP-034. None exhibit antimicrobial activity. With any new chemical entity, it is important to characterize metabolites and their impact on the pharmacokinetic (PK) profile as well as the absorption (as applicable), distribution, metabolism, and excretion (ADME) of the parent compound. In addition, the potential for drug-drug interactions (DDI) must be characterized. Clinically significant drug-drug interactions are well recognized with many antimicrobial drug classes (9), although novel tetracyclines pose a lower risk than other antimicrobials.

We report results from three studies in healthy subjects that investigated the ADME of i.v. eravacycline and the effect of a CYP3A4 inhibitor (itraconazole) and inducer (rifampin) on the PK of i.v. eravacycline.

## RESULTS

### Mass balance study.

Five male subjects were enrolled and completed the study. One subject did not complete the mass balance collection and was not included in the mass balance analyses but was included in the PK and safety analyses. Baseline demographics for all subjects are presented in Table 1.

**TABLE 1:** Baseline demographics

Parameter	ADME	DDI	
		Itraconazole	Rifampin
*N*	5	12	12
Age (yr)			
Mean	46.8	35.5	42.3
SD	1.8	12.5	10.4
Height (cm)			
Mean	176.4	171.6	174.1
SD	4.7	11.9	7.9
Weight (kg)			
Mean	87.1	69.7	77.5
SD	12.3	11.5	11.9
BMI (kg/m^2^)			
Mean	27.9	23.6	25.4
SD	2.7	3.0	2.2
Sex [no. (%)]			
Male	5 (100)	6 (50)	10 (83.3)
Female		6 (50)	2 (16.7)
Race [no. (%)]			
White	5 (100)	8 (66.7)	4 (33.3)
Black or African American		3 (25.0)	7 (58.3)
Pacific Islander		1 (8.3)	1 (8.3)

**FIG 1:**
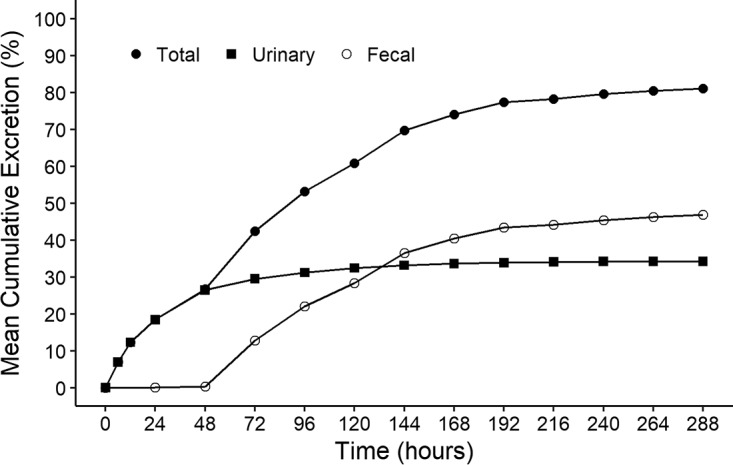
Cumulative total, urinary, and fecal excretion of radioactivity after a single 1-mg/kg i.v. dose of [^14^C]eravacycline.

**(i) Excretion of radioactivity.** Following i.v. administration of 60 mg [^14^C]eravacycline, 82.8% of the total radioactive dose was recovered in the urine and feces during the 288-h collection period (Fig. 1). Approximately half of the dose was recovered within the first 72 h. Since 47.8% of total radioactivity was recovered in the feces, biliary elimination should be considered the major route of elimination for eravacycline and/or its metabolites. Renal excretion accounted for approximately 34% of the dose.

**FIG 2:**
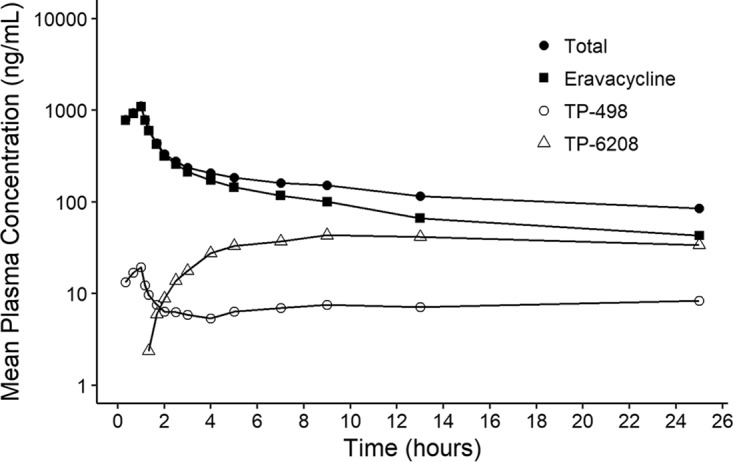
Plasma total, eravacycline, TP-498, and TP-6208 radioactivity after a single 1-mg/kg i.v. dose of [^14^C]eravacycline.

Whole-blood concentrations of total radioactivity reached maximum concentrations by the end of the i.v. infusion (range, 0.55 to 1 h) (Table 2). Thereafter, concentrations of total radioactivity declined in a biphasic manner and remained quantifiable for 49 to 73 h postdose. Similarly, plasma concentrations of total radioactivity reached maximum concentrations by the end of the infusion (0.55 to 1 h), but concentrations of total radioactivity declined in a triphasic manner with a number of secondary peaks (Fig. 2). Plasma concentrations remained quantifiable up to 241 h postdose in the majority of subjects.

**TABLE 2:** Geometric mean PK parameters for total radioactivity in whole blood and plasma after a single i.v. dose of eravacycline (*n* = 5)

Parameter	Geometric mean (%CV)	
	Whole blood	Plasma
*T*_max_^*a*^ (h)	1.0 (0.55, 1.0)	1.0 (0.55, 1.0)
*C*_max_ (ng equiv/ml)	911 (14.5)	893 (18.0)
AUC_0–last_ (ng equiv·h/ml)	8,440 (22.2)	12,100 (18.3)
AUC_0–24_ (ng equiv·h/ml)	4,880 (12.2)	4,420 (14.2)
MRT_0-last_ (h)	21.8 (20.7)	63.4 (8.9)
CL_R_^*b*^ (ml/min)	NC^*c*^	27.1 (17.6)

a Median (range).

b CL_R_, renal clearance.

c NC, not calculated.

**(ii) Pharmacokinetics.** Maximum plasma concentrations of eravacycline were reached by the end of the i.v. infusion (time to peak concentration [*T*_max_] ranged from 0.55 to 1 h) and then declined in a biphasic or triphasic manner; plasma concentrations were quantifiable for approximately 73 h postdose in the majority of subjects (Table 3). Geometric mean half-life (*t*_1/2_) was 18.2 h.

**TABLE 3:** Geometric mean PK parameters for eravacycline, TP-498, and TP-6208 in plasma after a single i.v. dose of [^14^C]eravacycline

Parameter	Value for [^14^C]eravacycline i.v. (*n* = 5) geometric mean (%CV)
Eravacycline	
*C*_max_ (ng/ml)	1,100 ± 13.5
AUC_0–∞_ (h·ng/ml)	4,380 ± 11.4 (*n* = 2)
*t*_1/2_ (h)	18.2 ± 5.0 (*n* = 2)
MRT_0-∞_ (h)	15.9 ± 8.9 (*n* = 2)
CL (ml/min)	228 ± 13.8 (*n* = 2)
CL_R_ (ml/min)	41.9 ± 16.6
*V*_ss_ (liters)	217 ± 4.9 (*n* = 2)
TP-498	
*C*_max_ (ng/ml)	20.4 ± 18.9
*T*_max_ (h)	1 (0.55–1)
CL_R_ (ml/min)	85.7 ± 35.5
TP-6208	
*C*_max_ (ng/ml)	42.8 ± 28.9
AUC_0–∞_ (h·ng/ml)	1780 ± 26.9 (*n* = 2)
*t*_1/2_ (h)	18.8 ± 17.8 (*n* = 2)

Maximum plasma concentrations of TP-498 occurred between 0.55 and 1 h and then declined in a monophasic manner, followed by a secondary rise in concentration or a plateau (Table 3). This prevented reliable determination of a terminal slope and half-life. TP-498 concentrations were quantifiable for 37 to 49 h after the i.v. dose. Plasma TP-6208 concentrations reached a maximum between 9 and 13 h postdose and then generally declined in a monophasic manner and remained quantifiable between 49 and 73 h postdose (Table 3). Geometric mean half-life was 18.8 h.

**(iii) Safety/tolerability.** Four (80%) subjects experienced at least 1 adverse effect (AE), and 3 experienced drug-related AEs. All 4 subjects reported gastrointestinal AEs, including abdomen discomfort or pain, constipation, diarrhea, or rectal hemorrhage. In addition, 1 subject reported chest pain and 2 reported headache. No serious or severe AEs and no discontinuations for AEs were recorded. No unexpected changes from baseline were observed for clinical laboratory tests, vital signs, physical examination, or electrocardiogram (ECG).

### Drug interaction studies.

Twelve subjects were enrolled in each study. Two subjects discontinued the itraconazole study; one discontinued due to an AE (presyncope) during the itraconazole treatment period, and one withdrew consent. Both subjects were excluded from the PK analysis population but included in the safety population. Baseline demographics for all subjects are presented in Table 1.

**FIG 3:**
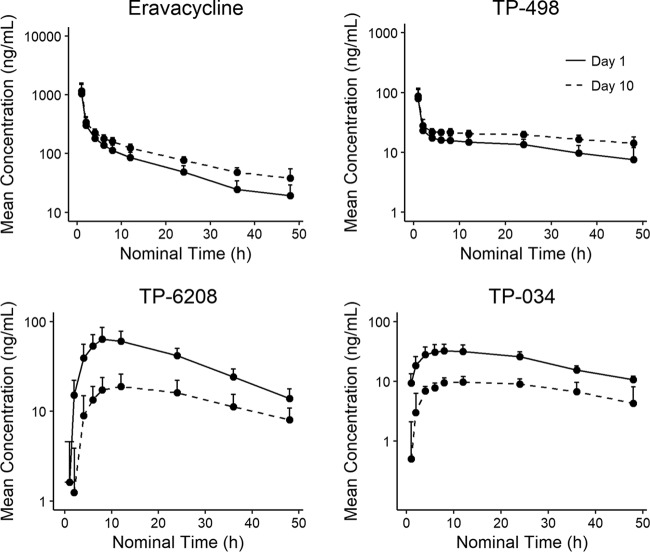
Mean eravacycline, TP-498, TP-6208, and TP-034 plasma concentrations over time on days 1 and 10 when administered with itraconazole.

**(i) Pharmacokinetic profile of eravacycline with and without itraconazole.** Following i.v. administration of eravacycline 1 mg/kg (day 1) and eravacycline plus itraconazole (day 10), eravacycline plasma versus time concentration curves were comparable (Fig. 3). The overall PK profiles were comparable between the treatments, although the area under the concentration-time curve from 0 h to the last time point (AUC_0_*_–t_*) and half-life were about 30% to 40% greater and CL was approximately 30% lower with eravacycline plus itraconazole on day 10 (Table 4). The ratio of geometric mean *C*_max_ for eravacycline plus itraconazole versus eravacycline was 107.9% and of geometric mean AUC_0–_*_t_* was 134.3% (Table 5). The 90% confidence interval (CI) for *C*_max_ fell within the standard bioequivalence criteria of 80% to 125%. Although the 90% CI for AUC_0–_*_t_* fell mostly outside the 80% to 125% bounds (124.1, 145.3), the distributions of AUC_0–_*_t_* for eravacycline and eravacycline plus itraconazole were comparable and overlapped significantly (day 1, 4,320 ± 1,250; day 10, 5,700 ± 1,090). Median *T*_max_ for eravacycline was 1 h after the start of the infusion on day 1 and day 10.

**TABLE 4:** Pharmacokinetic parameters for 1 mg/kg eravacycline administered i.v. on day 1 alone and day 10 with itraconazole (*n* = 10 subjects)

Parameter	Value (means ± SD) on day:	
	1	10
*C*_max_ (ng/ml)	1,100 ± 475	1,160 ± 420
AUC_0–_*_t_* (h·ng/ml)	4,320 ± 1,250	5,700 ± 1,090
*t*_1/2_ (h)	14 ± 2.4	19 ± 3.6
Clearance (ml/min/kg)	3.79 ± 1.03	2.57 ± 0.5
*V*_ss_ (liters/kg)	3.42 ± 0.73	3.37 ± 0.56

**TABLE 5:** Geometric mean PK parameters for eravacycline and metabolites after a single 1-mg/kg i.v. dose at day 10 (eravacycline plus itraconazole) and at day 1 (eravacycline alone)

Drug and parameter	Ratio of geometric mean (%)	90% CI for ratio (%)	*P* value^*a*^
Eravacycline			
*C*_max_ (ng/ml)	107.9	97.8, 119.0	0.189
AUC_0–_*_t_* (h·ng/ml)	134.3	124.1, 145.3	<0.0001
TP-498			
*C*_max_ (ng/ml)	105.3	94.9, 116.9	0.386
AUC_0–_*_t_* (h·ng/ml)	141.3	128.5, 155.5	<0.0001
TP-6208			
*C*_max_ (ng/ml)	29.7	26.4, 33.3	<0.0001
AUC_0–_*_t_* (h·ng/ml)	35.2	31.8, 39.1	<0.0001
TP-034			
*C*_max_ (ng/ml)	30.0	26.6, 33.7	<0.0001
AUC_0–_*_t_* (h·ng/ml)	31.1	26.1, 37.1	<0.0001

a Paired *t* test for comparison between day 10 and day 1 on log-transformed PK parameters. Ratio of geometric mean was calculated as day 10/day 1. The ratio and 90% CI are presented after back transformation to the original scale.

The ratios of geometric means for *C*_max_ and AUC_0–_*_t_* for the eravacycline metabolite TP-498 were generally comparable on day 1 and day 10, although a significant (*P* < 0.0001) difference was noted for AUC_0–_*_t_* (Table 5). For metabolites TP-6208 and TP-034, geometric mean values for *C*_max_ and AUC_0–_*_t_* were significantly (*P* < 0.0001) lower with eravacycline plus itraconazole (day 10) than with eravacycline alone (day 1).

**FIG 4:**
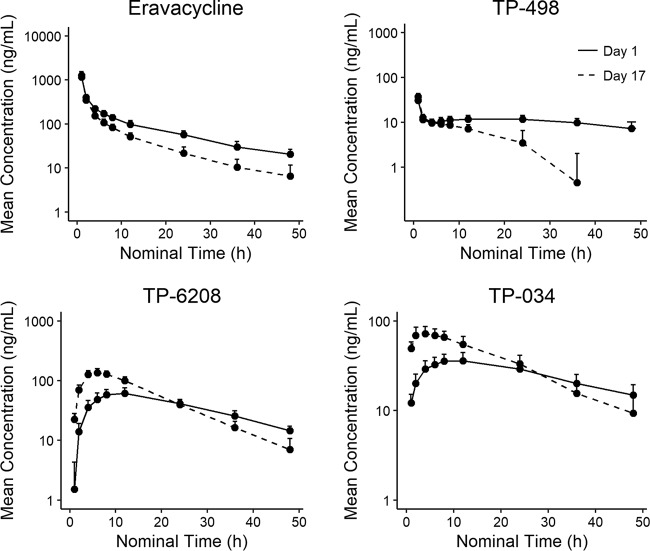
Mean eravacycline, TP-498, TP-6208, and TP-034 plasma concentrations over time on days 1 and 17 when administered with rifampin.

**(ii) Pharmacokinetic profile of eravacycline with and without rifampin.** Following i.v. administration of eravacycline at 1 mg/kg and eravacycline plus rifampin, plasma concentration curves for eravacycline on day 1 and day 17 showed a similar *C*_max_, but eravacycline was more rapidly cleared on day 17 in the presence of rifampin (Fig. 4). The eravacycline AUC was approximately 25% (AUC_0–24_) to 35% (AUC from time zero extrapolated to infinity [AUC_0–∞_]) lower and CL was approximately 50% greater with coadministration of rifampin (Table 6). The ratio of the geometric means of eravacycline plus rifampin versus eravacycline alone was 106.5% for *C*_max_ and 67.9% for AUC_0–_*_t_* (Table 6). The ratio was within the standard bioequivalence criteria of 80% to 125% for *C*_max._ The 90% CI of AUC_0–_*_t_* was outside the 80% to 125% bounds (63.5, 72.7). Although the distributions of AUC_0–_*_t_* for eravacycline and eravacycline plus rifampin were comparable, they overlapped less than with and without itraconazole (day 1, 4,100 ± 589; day 10, 3,090 ± 568).

**TABLE 6:** Pharmacokinetic parameters for 1 mg/kg eravacycline administered i.v. on day 1 alone and day 17 with rifampin^*a*^ (*n* = 12)

Parameter	Value (means ± SD) on day:	
	1	17
*C*_max_ (ng/ml)	1,170 ± 186	1,260 ± 281
AUC_0–∞_ (h·ng/ml)	5,380 ± 962	3,520 ± 726
AUC_0–24_ (h·ng/ml)	4,100 ± 589	3,090 ± 568
*t*_1/2_ (h)	15 ± 2.0	11 ± 2.3
CL (ml/min/kg)	3.20 ± 0.60	4.94 ± 1.08
*V*_ss_ (liters/kg)	2.85 ± 0.29	2.49 ± 0.51

a Rifampin at 600 mg was administered daily from day 8 to day 17.

Mean *C*_max_ and AUC_0–24_ for TP-498 were similar when eravacycline was dosed with or without rifampin. Mean *C*_max_ and AUC_0–24_ for TP-6208 and TP-034 were increased approximately 2-fold with rifampin. For TP-6208 and TP-034, *T*_max_ was observed earlier on day 17 (6 h for TP-6208, 4 h for TP-034) versus day 1 (12 h). The ratios of geometric means (90% CI) for *C*_max_ and AUC_0–_*_t_* for the eravacycline metabolite TP-498 were generally comparable at day 1 and day 17, although a significant (*P* < 0.0001) difference was noted for AUC_0–_*_t_* (Table 7). For metabolites TP-6208 and TP-034, geometric mean *C*_max_ and AUC_0–_*_t_* were significantly (*P* < 0.0001) higher with eravacycline plus rifampin (day 17) versus eravacycline alone.

**TABLE 7:** Geometric mean (%CV) PK parameters for eravacycline and metabolites after a single 1-mg/kg i.v. dose at day 17 (eravacycline plus rifampin) and day 1 (eravacycline alone)

Drug and parameter	Ratio (%) for^*b*^:		*P* value^*a*^
	Geometric mean	90% CI	
Eravacycline			
*C*_max_ (ng/ml)	106.5	98.0, 115.7	0.203
AUC_0–_*_t_* (h·ng/ml)	67.9	63.5, 72.7	<0.0001
TP-498			
*C*_max_ (ng/ml)	119.6	111.0, 128.8	0.0012
AUC_0–_*_t_* (h·ng/ml)	35.2	30.8, 40.1	<0.0001
TP-6208			
*C*_max_ (ng/ml)	222.3	203.7, 242.7	<0.0001
AUC_0–_*_t_* (h·ng/ml)	147.0	134.9, 160.3	<0.0001
TP-034			
*C*_max_ (ng/ml)	200.8	184.6, 218.3	<0.0001
AUC_0–_*_t_* (h·ng/ml)	138.3	127.5, 149.9	<0.0001

a Paired *t* test for comparison between day 17 and day 1 on log-transformed PK parameters.

b Ratio of geometric means was calculated as day 17/day 1. The ratio and 90% CI are presented after back transformation to the original scale.

**(iii) Safety/tolerability.** In the itraconazole study, 1 of 12 subjects (8.3%) experienced an AE during the eravacycline i.v. treatment period (nausea). Three of 11 subjects (27.3%) experienced four AEs during the itraconazole treatment period (headache, 2 subjects; rash, 1 subject; abdominal pain, 1 subject). One of 10 subjects (10.0%) experienced an AE during the eravacycline i.v. plus itraconazole treatment period (rash). All AEs were of mild or moderate severity. One treatment-emergent AE (TEAE; presyncope), experienced during the itraconazole treatment period and assessed by the investigator as not related to study drug, resulted in discontinuation of the subject from the study.

For the rifampin study, 3 of 12 (25.0%) subjects experienced an AE during the eravacycline i.v. treatment period (nausea, infusion pain, and pollakiuria, 1 subject each). One of 12 subjects (8.3%) experienced an AE during the rifampin treatment period (headache) and 1 (8.3%) during the eravacycline i.v. plus rifampin treatment period (headache). All TEAEs were mild in severity.

No serious AEs or discontinuations for AEs which were related to study drug were recorded in either study. No clinically significant abnormalities in vital signs, laboratory values, physical examination, or ECG were observed in either study.

## DISCUSSION

Intravenous eravacycline is undergoing clinical development as treatment for serious bacterial infections, including cIAI. As part of a development program for new chemical entities, the Food and Drug Administration specifies that studies be conducted to elucidate ADME via a mass balance study in healthy subjects (10). Further, based on evidence from preclinical studies of the potential for clinically significant drug-drug interactions, clinical studies are required to examine this potential in healthy subjects (11).

In the mass balance study, the majority of total radioactivity from [^14^C]eravacycline was recovered in the feces, suggesting biliary/fecal elimination as the major route of excretion for eravacycline and its metabolites after i.v. administration. Importantly, dosage adjustment is unlikely to be needed in subjects with renal insufficiency, as confirmed by a study in subjects with end-stage renal disease (12).

Whole-blood-to-plasma concentration ratios suggest preferential distribution of eravacycline and/or its metabolites to the cellular component of whole blood following i.v. administration. Of note, less than 100% of radioactivity was recovered primarily due to sequestration of eravacycline in the bone and other tissues, as was demonstrated in a study of the PK and tissue distribution of multiple doses of eravacycline in rabbits (13). These results are similar to what has been reported with other tetracyclines (14). The volume of distribution (217 liters) with i.v. eravacycline in healthy subjects was greater than that of extracellular fluid, which suggests distribution beyond the central compartment and may be due in part to the distribution and absorption in bone.

Following i.v. administration of [^14^C]eravacycline, TP-498 concentrations peaked rapidly, with a *T*_max_ of 1.0 h. Peak concentrations for the metabolites were lower than that for eravacycline; geometric mean *C*_max_ and AUC_0–_*_t_* were 53.9-fold and 13.5-fold lower for TP-498, suggesting limited formation. Formation of TP-6208 was slow, with a *T*_max_ of 13 h. The geometric mean *C*_max_, AUC_0–_*_t_*, and AUC_0–∞_ for TP-6208 were 25.7-fold, 2.9-fold, and 2.5-fold lower than those of eravacycline.

In the drug-drug interaction studies, mean AUC_0–_*_t_* and half-life were increased approximately 30% to 40% after a concomitant dose of i.v. eravacycline and itraconazole, and CL was approximately 30% lower. This interaction is unlikely to be of clinical significance, since the resulting exposures are well within the range shown to be safe and tolerable in clinical trials. Concomitant administration of rifampin with i.v. eravacycline resulted in a reduction in total eravacycline exposure (AUC) of approximately 25% to 35% and an increase in CL of approximately 50%. Since the reduced exposure could lead to decreased efficacy, the dose of eravacycline should be increased to 1.5 mg/kg q12h when coadministered with a strong CYP3A inducer, such as rifampin or phenytoin. Population-PK modeling suggests that this dose adjustment will result in day 1 and steady-state exposures that closely approximate those for the 1-mg/kg q12h dose in the absence of CYP3A inducers. The PK profile of i.v. eravacycline across these 3 studies was otherwise consistent with results from other studies of eravacycline in healthy volunteers (3, 4).

The safety/tolerability profile was similar across all 3 studies, which were conducted with the same 1-mg/kg i.v. dose of eravacycline infused over 60 min. No unexpected safety/tolerability concerns were identified with i.v. eravacycline in these 3 studies in healthy subjects. The 1-mg/kg dosage regimen is the same as that used in phase 2 and 3 clinical studies of i.v. eravacycline for cIAI (7, 8).

The ADME profile of the eravacycline metabolites TP-498, TP-6208, and TP-034 suggests that they will have little impact on the safety/tolerability profile of eravacycline and should not result in any significant CYP3A4-mediated drug-drug interactions. Previous studies have shown no *in vitro* antimicrobial activity for any of these compounds. In summary, i.v. eravacycline exhibited an ADME profile that was consistent with preclinical studies showing extensive tissue distribution but mostly fecal/biliary excretion. Results of coadministration of eravacycline with an inducer and an inhibitor of CYP3A4 indicate a low risk for clinically significant drug-drug interactions, except with strong CYP3A inducers, when a dose adjustment is warranted to ensure sufficient exposure.

## MATERIALS AND METHODS

All three studies were conducted in accordance with the International Conference on Harmonization Guidelines for Good Clinical Practice (15) and the Declaration of Helsinki regarding the treatment of human subjects. The study protocols and informed consent forms were reviewed and approved by an appropriate Ethics Committee/Institutional Review Board. All subjects provided written informed consent prior to participation in any study procedures.

### Mass balance study.

**(i) Materials.** Eravacycline (lot number CMLW-201/12-TP6) was manufactured by Metrics, Inc., Greenville, NC, USA, and supplied to Selcia Limited, Essex, UK. [^14^C]Eravacycline (batch number 7964MXM001-3) was manufactured and supplied by Selcia Limited, Essex, UK, on behalf of Quotient Clinical. Radiochemical purity was determined by reference standards provided by Tetraphase Pharmaceuticals and was 89.3%, 88.6%, and 86.7% for eravacycline, TP-498, and TP-6208, respectively.

**(ii) Study design.** This was a single-center, open-label study of a single i.v. dose of [^14^C]eravacycline to assess the mass balance recovery and PK of eravacycline. The primary objectives were to determine mass balance recovery, determine routes and rates of excretion, provide metabolite profiling and structural identification, and assess the PK of radioactivity in plasma and whole blood. Subjects were screened for inclusion in the study, including a detailed medical history, physical examination, and laboratory evaluations, in the 28 days before dosing. Eligible subjects were admitted to the clinical study unit at approximately 21:00 on the evening prior to study drug administration (day −1) and remained on site until 240 h postdose for collection of urine, feces, and blood samples. After an overnight fast, each subject received 60 mg [^14^C]eravacycline containing not more than 3.8 MBq (105 μCi) ^14^C as a single i.v. infusion over 60 min.

**(iii) Subject selection.** Healthy male subjects, ages 30 to 65 years with body mass index (BMI) of 18.5 to 32.0 kg/m^2^, were eligible. Subjects were excluded for a history of drug or alcohol abuse in the previous 2 years; regular alcohol consumption (>21 U/week) or current or recent smoking history; clinically significant laboratory abnormalities; history of cardiovascular, renal, hepatic, chronic respiratory, or gastrointestinal disease; hypersensitivity to tetracyclines or related compounds; or use of any concomitant drugs within 14 days of the study.

**(iv) Determination of radioactivity.** Whole-blood and plasma samples were collected predose, at 20, 40, and 60 min after the start of the infusion, and at 10, 20, and 40 min and 1, 1.5, 2, 3, 4, 6, 8, 12, 24, 36, 48, 72, 96, 120, 144, 168, 192, 216, and 240 h after the completion of the infusion to measure eravacycline, TP-498, and TP-6028 concentrations. Blood samples (4 ml) for whole-blood assessments were collected into sodium heparin (polypropylene) tubes. Samples were inverted at least 5 times and then stored at 2°C to 8°C until being shipped for analysis. Blood samples (6 ml) for plasma assessments were collected into sodium heparin (polypropylene) tubes. Samples were inverted at least 5 times and then placed on ice until centrifugation. Samples were centrifuged at 1,500 × *g* for 10 min at 4°C within 30 min of sample collection. The resultant plasma from each sample was transferred into a polypropylene tube and contained at least 2 ml of plasma. Samples were frozen within 30 min of centrifugation and stored at approximately −20°C until being shipped for analysis. Plasma and whole-blood concentrations of total radioactivity were determined using an analytical method at Quotient Bioresearch (Rushden, Northamptonshire, UK). The lower limit of quantification (LLOQ) for total radioactivity was 11 to 13 ng equivalents (equiv)/ml in plasma and 41 to 46 ng equiv/g in whole blood.

**(v) Excretion of radioactivity.** Mass balance was determined from the incremental amount and cumulative amount excreted in urine, feces, and urine and feces combined. Amount excreted and amount excreted expressed as a percentage of administered dose were determined for urine, feces, and total samples.

**(vi) Study assessments.** PK parameters were determined for total radioactivity in plasma and whole blood. Eravacycline, TP-498, and TP-6208 concentrations in plasma were quantified using a validated assay with internal standards. The following PK parameters were determined: *C*_max_, *T*_max_, time from dosing before the first quantifiable concentration in a concentration versus time profile (*T*_lag_), AUC_0–*t*_, AUC_0–24_, AUC_0–∞_, the percentage of AUC_0–∞_ extrapolated beyond the time of the last measurable concentration (AUC_%extrap_), the slope of the apparent terminal phase (lambda-z), the apparent terminal half-life (*t*_1/2_), the mean residence time estimated between time zero and infinity (MRT_0-∞_), the apparent volume of plasma cleared of analyte per unit time (CL), and the volume of distribution of analyte at steady state (*V*_ss_).

Plasma concentrations of eravacycline, TP-498, and TP-6208 were measured by liquid chromatography with tandem mass spectrometry (LC-MS). The LLOQ was 5 ng/ml for eravacycline, TP-498, and TP-6208. The calibration ranges for eravacycline, TP-498, and TP-6208 in human urine were 50 to 10,000 ng/ml, 50 to 1,000 ng/ml, and 20 to 1,000 ng/ml, respectively. The calibration ranges for eravacycline, TP-498, and TP-6208 in human plasma were 5 to 500 ng/ml.

Safety was assessed from adverse events (AEs), physical examinations, laboratory safety tests (hematology, coagulation, clinical chemistry, and urinalysis), vital signs, ECGs, and body weight.

**(vii) Metabolite profiles.** Plasma samples for metabolite identification were collected predose and at 20 min and 2, 4, 12, 24, 48, 96, and 168 h after the completion of the infusion. Urine samples were collected at 0- to 6-, 6- to 12-, and 12- to 24-h intervals, and feces samples were collected predose and at 24-h intervals through day 11 or discharge for metabolite profiling.

Blood samples were collected into sodium heparin (polypropylene) tubes and centrifuged at 1,500 × *g* for 10 min at 4°C within 30 min of sample collection. Plasma samples were stored at −70°C until shipment. Urine samples were collected into individually labeled polypropylene containers and then weighed. Samples that were not processed immediately were stored at 2°C to 8°C. For each collection interval, 2%, vol/vol, of the urine sample was aliquoted for metabolite profiling and identification. The urine aliquots were stored at −70°C until shipment for analysis. Pooled feces samples were collected from each subject for each collection interval and homogenized. Immediately after homogenization, two 50-g aliquots were stored at −70°C or below until shipment. Metabolite profiling and identification were performed on plasma, urine, and feces samples by Frontage Laboratories (Exton, PA).

**(viii) Study analysis.** Descriptive statistics (mean, median, standard deviation [SD], percent coefficient of variation [%CV], minimum, maximum, number, geometric mean, and geometric %CV) were calculated for all concentration data. Pharmacokinetic parameters were calculated using Phoenix WinNonlin v6.3 (Certera USA, Inc.).

### Drug interaction studies.

Two single-center, open-label studies were conducted to evaluate the effects of itraconazole (CYP3A4 inhibitor) and rifampin (CYP3A4 inducer) on the pharmacokinetics of i.v. eravacycline in healthy subjects.

Eravacycline for i.v. administration was supplied as a sterile injectable lyophilized powder in a 10-ml vial. The powder was reconstituted with sterile water and further diluted with sterile 0.5% normal saline to generate 0.3-mg/ml eravacycline solutions for 1 mg/kg i.v. infusions. Itraconazole and rifampin were obtained as marketed formulations of each product.

**(i) Subject selection.** Healthy subjects, ages 18 to 55 years with BMI of 18 to 30 kg/m^2^, were enrolled if they met all eligibility criteria. Eligible subjects had negative alcohol, tobacco, and drug tests at screening and had no clinically significant medical conditions that could interfere with the conduct of the studies. Subjects were excluded for the presence of clinically significant ECG abnormalities, elevated blood pressure or heart rate, liver function tests greater than 1.5 times the upper limit of normal, or evidence of renal impairment (estimated glomerular filtration rate of <60 ml/min/1.73 m^2^). Subjects were excluded for known allergy to tetracyclines or related compounds or to itraconazole or rifampin. Subjects also were excluded for use of alcohol or nicotine within 48 h of dosing, history of alcoholism or drug abuse within 2 years, or use of any concomitant medications within 7 days or 5 half-lives. Females were required to be surgically sterile or have a negative pregnancy test at screening and baseline. Male subjects were required to use a barrier contraceptive method during drug administration until 7 days after the end of the study.

**(ii) Study design.** Each study included a 28-day screening period, a 13-day treatment period (19 days for rifampin), and an end-of-study visit 2 weeks after the last dose of study drug. Eravacycline was administered in the morning as a 1-mg/kg i.v. infusion over 60 min on day 1 with a second dose on day 10 (itraconazole study) or day 17 (rifampin study). For the itraconazole study, 200 mg oral itraconazole was administered q12h on day 8 and q24h on days 9 and 10. For the rifampin study, 600 mg oral rifampin was administered q24h from day 8 to day 17. All study treatments were administered in the fasting state.

**(iii) Study assessments.** Blood samples for PK analysis of eravacycline and its metabolites, TP-498, TP-6208, and TP-034, were collected predose and 1, 2, 4, 6, 8, 12, 24, 36, and 48 h after the start of the i.v. infusion on day 1 and day 10 (itraconazole) or day 1 and day 17 (rifampin). Safety assessments included AEs, clinical laboratory safety tests (hematology, serum chemistry, electrolytes, coagulation, urinalysis, and lipid profile), vital signs, physical examinations, and 12-lead ECG.

Concentrations of eravacycline, TP-498, and TP-6208 in human plasma samples were measured by LC-tandem MS after solid-phase extraction over a calibration range of 5.00 to 500 ng/ml. The LLOQ for eravacycline, TP-498, TP-6208, and TP-034 in human plasma was 5.00 ng/ml. Samples with eravacycline initial concentrations above the upper limit of quantification (500 ng/ml) were diluted appropriately with control blank human plasma and reanalyzed. There was no interference seen when human plasma was fortified with eravacycline, TP-498, TP-6208, and either itraconazole or rifampin.

**(iv) Pharmacokinetic analysis.** PK assessments included *C*_max_, *T*_max_, AUC_0–24_, AUC_0–_*_t_*, *t*_1/2_, CL, and *V*_ss_. Actual sampling time was used for PK calculations. PK parameters were derived by noncompartmental analysis. Descriptive statistics were calculated for analyte concentrations at each evaluation time point and for all quantitative PK parameters of eravacycline, TP-498, TP-6208, and TP-034 for day 1 and day 10 (day 17 for rifampin). The effect of itraconazole or rifampin on the PK profile of i.v. eravacycline was evaluated by comparing log-transformed PK parameters (*C*_max_ and AUC_0–_*_t_*) associated with the administration of study drug on day 1 and day 10 (itraconazole) or day 1 and day 17 (rifampin) using paired *t* tests. The ratio and 90% confidence interval (CI) were presented after back transformation to the original scale. Pharmacokinetic variables were calculated using WinNonlin Professional.
